# Supplementary material for: Causal Relationship between Aging and Anorexia Nervosa: A White-Matter-Microstructure-Mediated Mendelian Randomization Analysis
Source: Biomedicines. 2024 Aug 16;12(8):1874. doi: 10.3390/biomedicines12081874 (PMC11351342; doi:10.3390/biomedicines12081874)
Supplement: Supplementary file 1 [file biomedicines-12-01874-s001.zip › Supplementary Figure/Figure S4/Singe SNP plot of MR analysis for TL to ubm-b-1731.pdf]

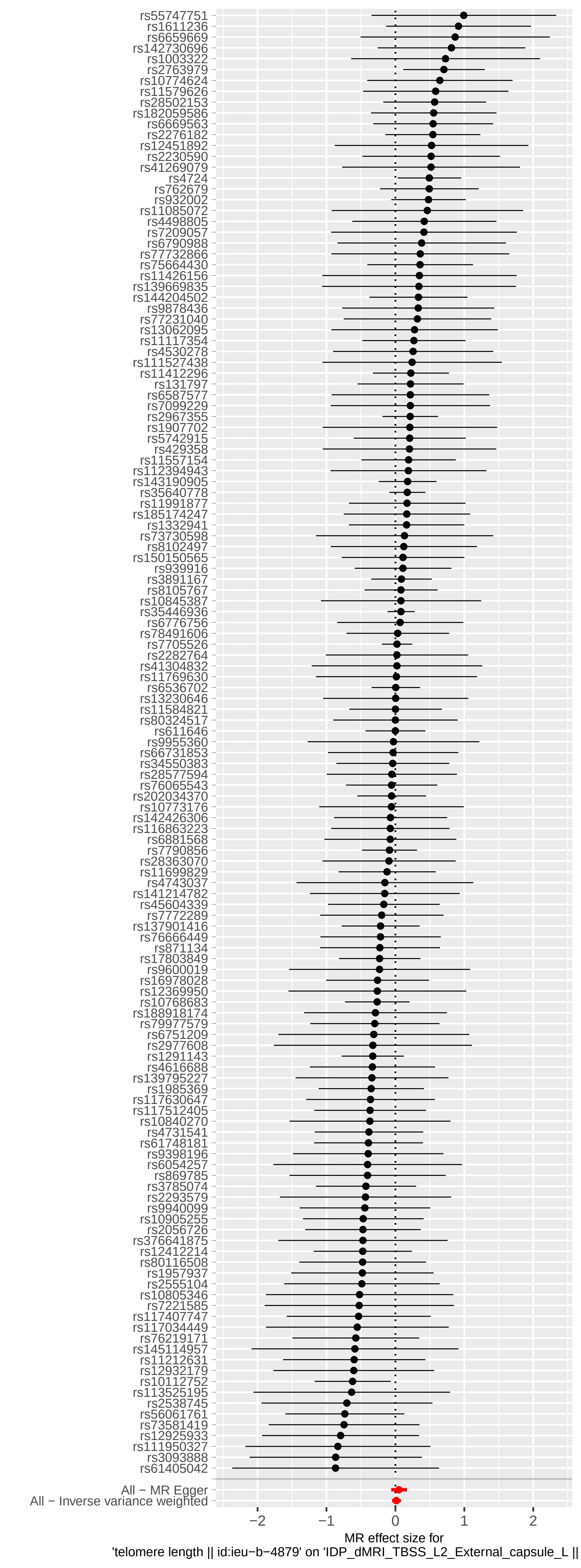
rs55747751
rs1611236
rs6659669
rs142730696
rs1003322
rs2763979
rs10774624
rs11579626
rs28502153
rs182059586
rs6669563
rs2276182
rs12451892
rs2230590
rs41269079
rs4724
rs762679
rs932002
rs11085072
rs4498805
rs7209057
rs6790988
rs77732866
rs75664430
rs11426156
rs139669835
rs144204502
rs9878436
rs77231040
rs13062095
rs11117354
rs4530278
rs111527438
rs11412296
rs131797
rs6587577
rs7099229
rs2967355
rs1907702
rs5742915
rs429358
rs11557154
rs112394943
rs143190905
rs35640778
rs11991877
rs185174247
rs1332941
rs73730598
rs8102497
rs150150565
rs939916
rs3891167
rs8105767
rs10845387
rs35446936
rs6776756
rs78491606
rs7705526
rs2282764
rs41304832
rs11769630
rs6536702
rs13230646
rs11584821
rs80324517
rs611646
rs9955360
rs66731853
rs34550383
rs28577594
rs76065543
rs202034370
rs10773176
rs142426306
rs116863223
rs6881568
rs7790856
rs28363070
rs11699829
rs4743037
rs141214782
rs45604339
rs7772289
rs137901416
rs76666449
rs871134
rs17803849
rs9600019
rs16978028
rs12369950
rs10768683
rs188918174
rs79977579
rs6751209
rs2977608
rs1291143
rs4616688
rs139795227
rs1985369
rs117630647
rs117512405
rs10840270
rs4731541
rs61748181
rs9398196
rs6054257
rs869785
rs3785074
rs2293579
rs9940099
rs10905255
rs2056726
rs376641875
rs12412214
rs80116508
rs1957937
rs2555104
rs10805346
rs7221585
rs117407747
rs117034449
rs76219171
rs145114957
rs11212631
rs12932179
rs10112752
rs113525195
rs2538745
rs56061761
rs73581419
rs12925933
rs111950327
rs3093888
rs61405042
All − MR Egger
All − Inverse variance weighted
−2
−1
0
1
2
MR effect size for
'telomere length || id:ieu−b−4879' on 'IDP_dMRI_TBSS_L2_External_capsule_L ||
